# Optimization and computational studies evaluating molecular dynamics of EDA cored polymeric dendrimer

**DOI:** 10.1038/s41598-020-77540-x

**Published:** 2020-12-15

**Authors:** Malvika Chawla, R. D. Kaushik, Jaspal Singh, Manila

**Affiliations:** Department of Chemistry, Gurukul Kangri Vishwavidyalaya, Haridwar, Uttrakhand India

**Keywords:** Molecular medicine, Chemistry, Nanoscience and technology

## Abstract

In this work we report the results acquired from molecular dynamics simulations as well as the optimization of different generations of polyamidoamine dendrimer. The analysis data revealed synthesized dendrimer as a suitable nanostructured candidate suitable for neutral as well as charged molecule delivery due to the presence of both electrostatic potential and van der Waals forces. The methyl ester terminating groups of half-generation dendrimers with characteristic IR peaks for carbonyl at 1670.41 cm^−1^ tends to shift to 1514.17 cm^−1^ on conversion to amide group of full-generation dendrimer. The study includes the usage of detailed analysis, demonstrating how molecular dynamics affect the dendrimer complexation. The present investigations provide an unprecedented insight into the computational and experimental system that may be of general significance for the clinical application of dendrimers.

## Introduction

The modern age revolutionized the foundation of a new era of molecular polymers i.e. polymeric dendrimers. The nanostructured polymers are repetitively bifurcated morphological spherical molecules with distinguishing chemical elegance, pertaining to flexible, versatile and promising molecular representatives^1,2^. The polymers are constructed via a repetitive successive process based primarily on two different approaches: the divergent^3,4^ and the convergent^5–7^. Former one deals with the addition of monomers to the core forming layers and the later one starts from the periphery to the core. Since the initial reports of dendrimers various systems have been reported and received widespread attention. In addition, due to structural and chemical diversity, they have produced several opportunities for modification. They have come up as an excellent platform for terminal attachment of multiple close-packed functionalities, as well as cavities around the focal core permit various opportunities^8,9^.

The multifunctional hyperbranched analogues with tunable physicochemical attributes of the Tomalia’s polyamidoamine^10^ make them promising candidates for poorly soluble drugs^11–32^ such as fluorine^33^, complexes with metal ions^34^, catalysis^35,36^, gene delivery^37,38^, drug delivery^39^, sensors, and many other. The influence on chemical and physical properties of dendritic polymers by peripheric moieties have been confirmed by an assortment of reports. The peripheral functionalities are also responsible for impacting the stability, solubility, viscosity, flexibility, aggregation and chemical reactivity as well as spatial and surface shape of the dendrimer^40^. The terminal impact on molecular properties increases with the exponential growth of dendrimer generation. Moreover, a molecule periphery functionalization is the most promising and straightforward option in terms of creating novel dendrimer properties.

In the direction towards the contemplation of molecular dynamics inclusive of molecular interaction at the molecular level, the molecular modeling emerged as a potential means. The algorithmic methodology provides better and broader molecular perception, making molecule designing effective and simpler. The hyperbranched dendritic polymer encompassing repetitious structure can be competently taken advantage of in designing novel porters for both therapeutic and diagnostic agents. The studies discussing the optimization of its properties in various solvents, interaction with drugs, nucleic acids, proteins and lipid membranes have been studied. The pondered researches have brought characteristic features such as dendrimer size and surface to light, that can be altered to augment the performance of anti-TB^41^, anti-cancer^42–44^ drugs and many other. There are reports of dendritic geometry optimization using forcefield such as AMBER^45,46^, CHARMM^47–49^, GROMOS^50^, MARTINI^51–53^ CVFF^54,55^, OPLS^56,57^ and DREIDING Force^58^.

The knowledge of molecular dynamics by computational studies have advanced experimental work. For enhanced envision and insight of dendrimers and their interactions, computational designs provide sustenance to ameliorate efficiency. This investigational approach is converged on the optimization and application of molecular modeling to analyze the layout of dendrimers. The emphasis has been made to improve the efficacy of this class of molecules. In this paper, dendrimer molecular models were parameterized using the UFF forcefield and molecular dynamic atomistic simulation approaches have been employed. Simultaneously, the dendritic polymers were optimized at laboratory scale. To meet the augmenting requirement of dendrimers for multiple applications, the co-application of computational and experimental approach can significantly enhance the clinical application of dendrimers.

## Results

### Evaluation of computational studies

**Figure 1:**
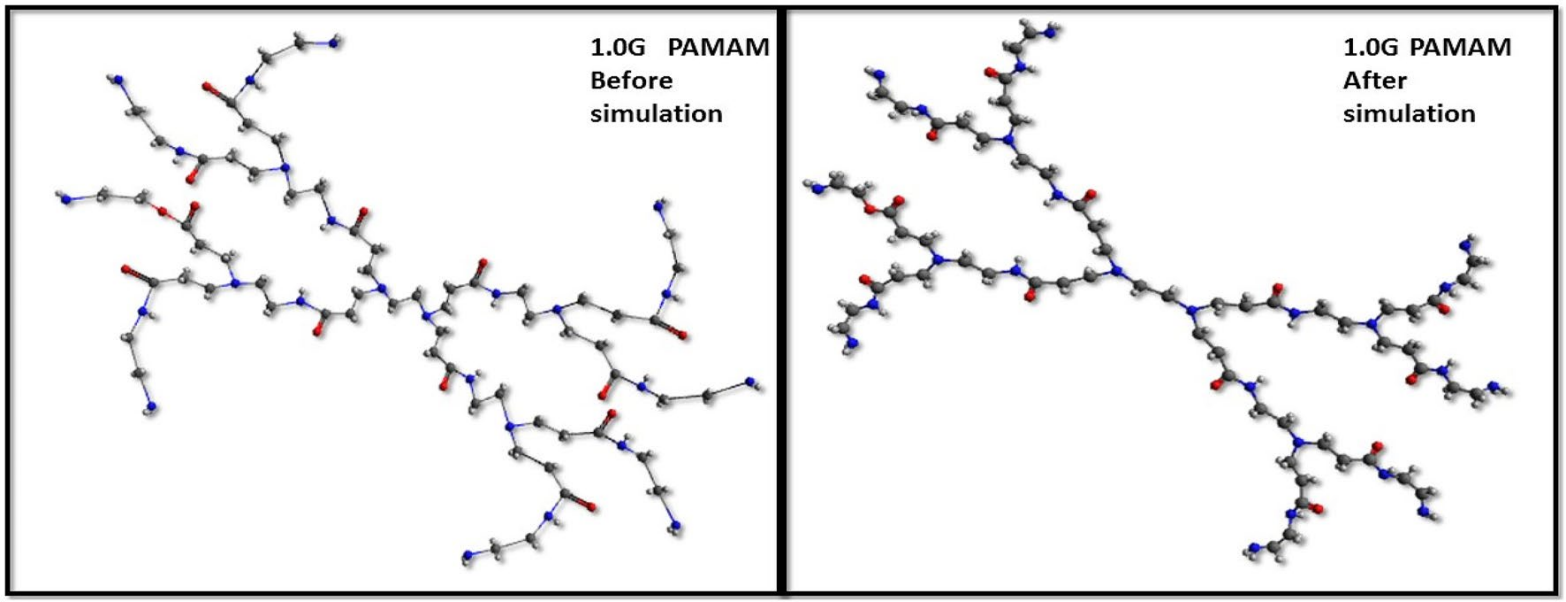
Illustrations showing parameterized simulations on 1.0G PAMAM (Avogadro 1.1.1, http://avogadro.cc).

Essentially like all molecules, the PAMAM (Polyamidoamine) dendrimer structure shows correspondence with properties and its utilization. The synthesis of highly branched structures with potential practice is directly or indirectly dependent on intrinsic features such as molecular weight, van der wall forces. The UFF elucidates the forces workable for simulation of atoms within dendrimer structure, exhibiting the sum of molecular properties as well as electrostatic and van der Waals interactions, Fig. 1.

**Figure 2:**
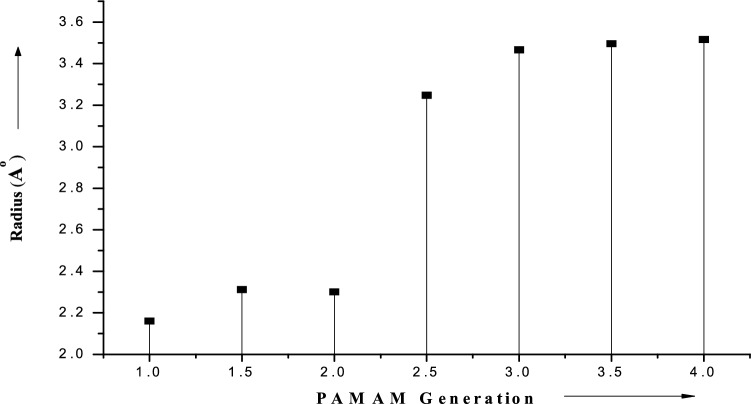
Polyamidoamine generation dependence of the average radius between central atom of dendrimer (OriginPro 8 SR0, v8.0724 (B724), OriginLab Corporation: http://www.OriginLab.com).

In this study we also contemplate the effect of size of dendrimer generation on the conformational characteristics. The deportment of the simulations conducted divulge the augment in average radius corresponding to the increased dendrimer generation. Consequently, the increment in radius of central atom magnifies the distance among atoms. Hence, these alterations toil synergistically with the entrapment or encapsulation of moiety by dendrimer. The simulation studies of PAMAMs overseen in this communication (Fig. 2) manifest the use of high generation dendrimer molecules as a competent approach for drug delivery.

**Figure 3:**
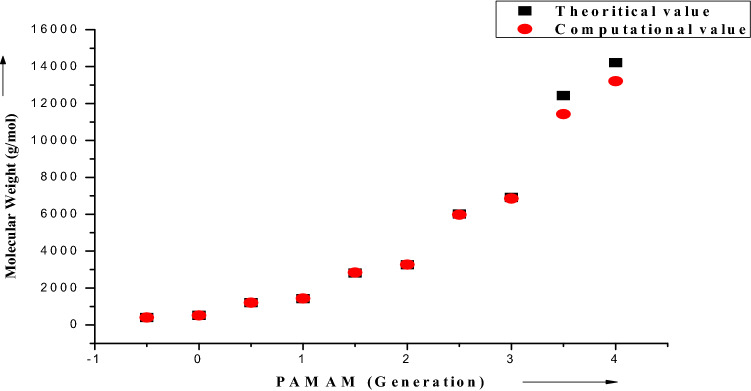
Graphical plot showing alter in molecular weight obtained from theoretical and computational studies of dendrimers (OriginPro 8 SR0, v8.0724 (B724), OriginLab Corporation: http://www.OriginLab.com).

Taking into cognizance, both experimental and simulation studies, the moderate size of the dendrimer concede them to acquit as soft deformable particles rather than compact spheres, reproducing them as an attributable candidate for drug delivery. This is conclusively confirmed in the present study. Comparison between the computational and theoretically calculated molecular weight are shown in Table 1 and Fig. 3. The inferences distinctly substantiate the close agreement between computational and theoretical data.

**Table 1:** Molecular dynamics of different generation of polyamidoamine dendrimer.

Generation	Number of end groups	Theoretical molecular weight (g/mol)	Calculated Molecular weight post simulation (g/mol)
− 0.5	4	405	404.455
0.0	4	517	516.681
0.5	8	1205	1205.395
1.0	8	1430	1430.831
1.5	16	2807	2836.312
2.0	16	3256	3266.065
2.5	32	6011	5974.471
3.0	32	6909	6846.870
3.5	64	12,424	11,422.589
4.0	64	14,215	13,212.462

**Figure 4:**
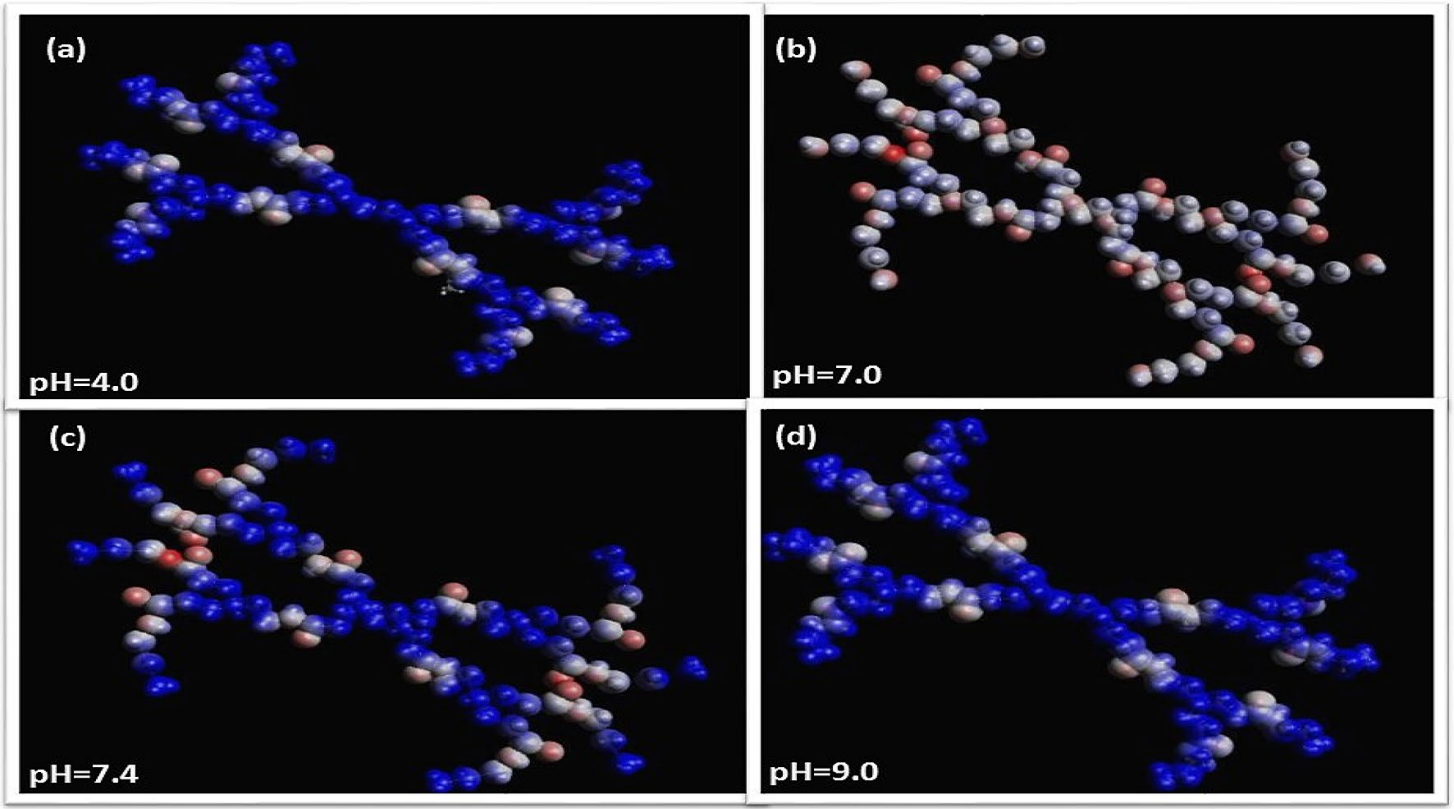
Comparison of Van der Walls effect on 1.0G polyamidoamine generation at (**a**) pH = 4.0, (**b**) pH = 7.0, (**c**) pH = 7.4 and (**d**) pH = 9.0 (Blue region: Van der Waals sphere; Other region: Electrostatic potential) (Avogadro 1.1.1, http://avogadro.cc).

The detailed effect of the different degree of protonation (i.e., different pH values), was studied by executing simulations of molecules at four different pH levels. The effect of pH was scrutinized using molecular editor, Avogadro, that concede the pH disposition of the molecular environment. All MD simulations were carried out in aqueous solutions under realistic requisites of different pH levels. The assessment of van der Waals spheres was found to be significant in the characteristic determination of dendrimers. These spheres stipulate information in correlation with internal cavities^59^, which can be further utilized for the determination of number of molecules dendrimers will be competent to sustain (Fig. 4). The pretense of both electrostatic potential and van der Waals region conjointly validates the dendrimer efficient for binding neutral as well as charged molecule.

### Evaluation of dendrimer

**Figure 5:**
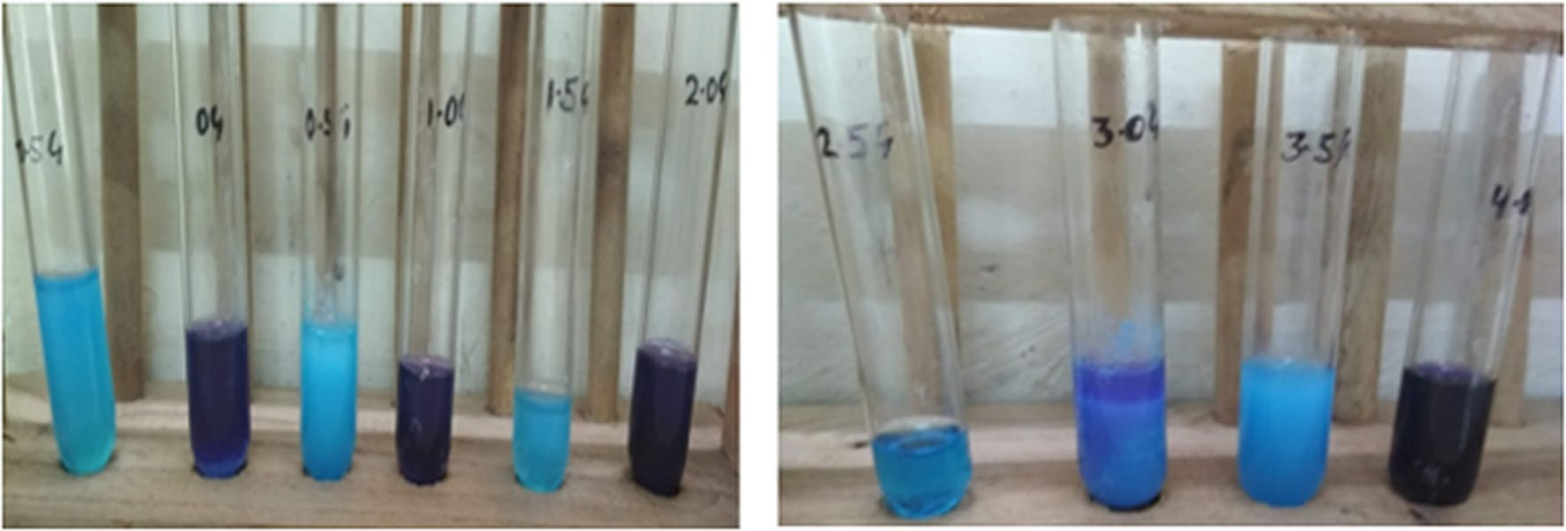
Copper sulphate test results of different generations of polyamidoamine.

Progress of synthesis and differentiation of full and half-generation of dendrimers were confirmed spectrophotometrically. The synthesis of dendrimers reported here employed the usage of ethylenediamine and methyl acrylate, where, former act as initiator core and later as a repeating unit. The yields of 1.0G, 2.0G, 3.0G and 4.0G were reported to be 95, 92, 84 and 81%, respectively: and 0.5G, 1.5G, 2.5G and 3.5G were 90, 84, 81 and 72%, respectively. The copper sulfate color chelation reactions affirmed the completion of each synthetic step. The copper sulfate on reaction with terminal amine group gave deep blue color signifying the presence of half generation, whereas, reaction with terminal carboxyl group gave purple color distinctly affirming the presence of full generation, Fig. 5.

The inquisition detailed here affirm the termination of each synthetic step included in the preparation of the PAMAM dendrimer. A startling change in color was noticed after admixture of carboxyl/amine terminated dendrimer with copper sulfate solution. The observation led researchers to conclude the formation of complex on combination of copper with terminal groups. Consequently, the blue precipitate is formed due to the reaction of hexaaquacopper(II) complex ion –[Cu(H_2_O)_6_]^2+^ present in copper sulphate solution with terminal amine group. Similarly, admixture of carboxyl terminated dendrimer and copper sulfate solution produced the purple color precipitates.

**Figure 6:**
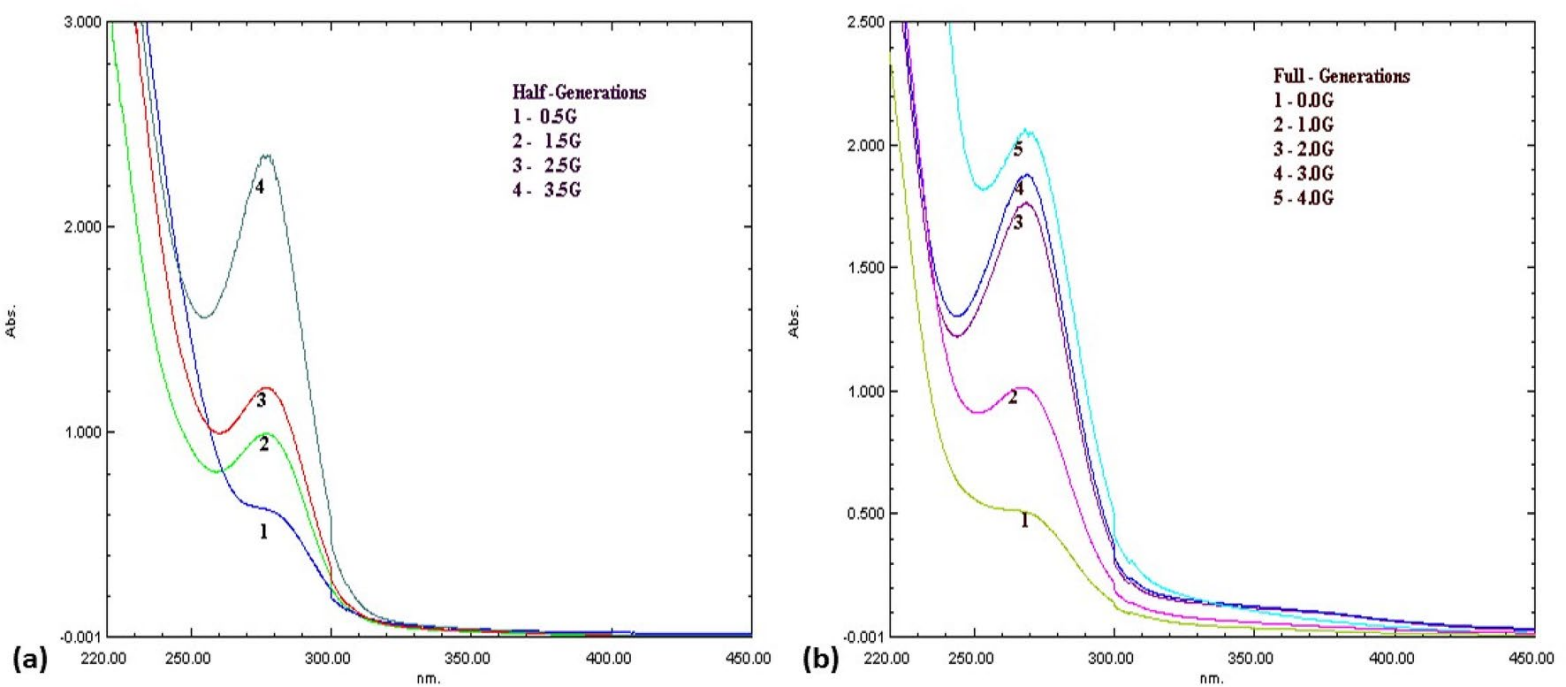
UV–Vis absorption spectra obtained from different generations of PAMAM dendrimer in methanol (**a**) half generation; (**b**) full generation.

The synthesis of dendrimer was overseen by usage of UV–Visible spectroscopy. The variation in λmax values perceived from half-generation to full generation i.e. from 290 to 270 nm impart the structural modification of PAMAM (Polyamidoamine) dendrimers, as shown in Fig. 6. The proportional correlation between absorption and number of chromophoric units explicate the alter in the intensity of the absorption band with the growing generation. This manifests the conformation of synthesis of dendrimers.

**Figure 7:**
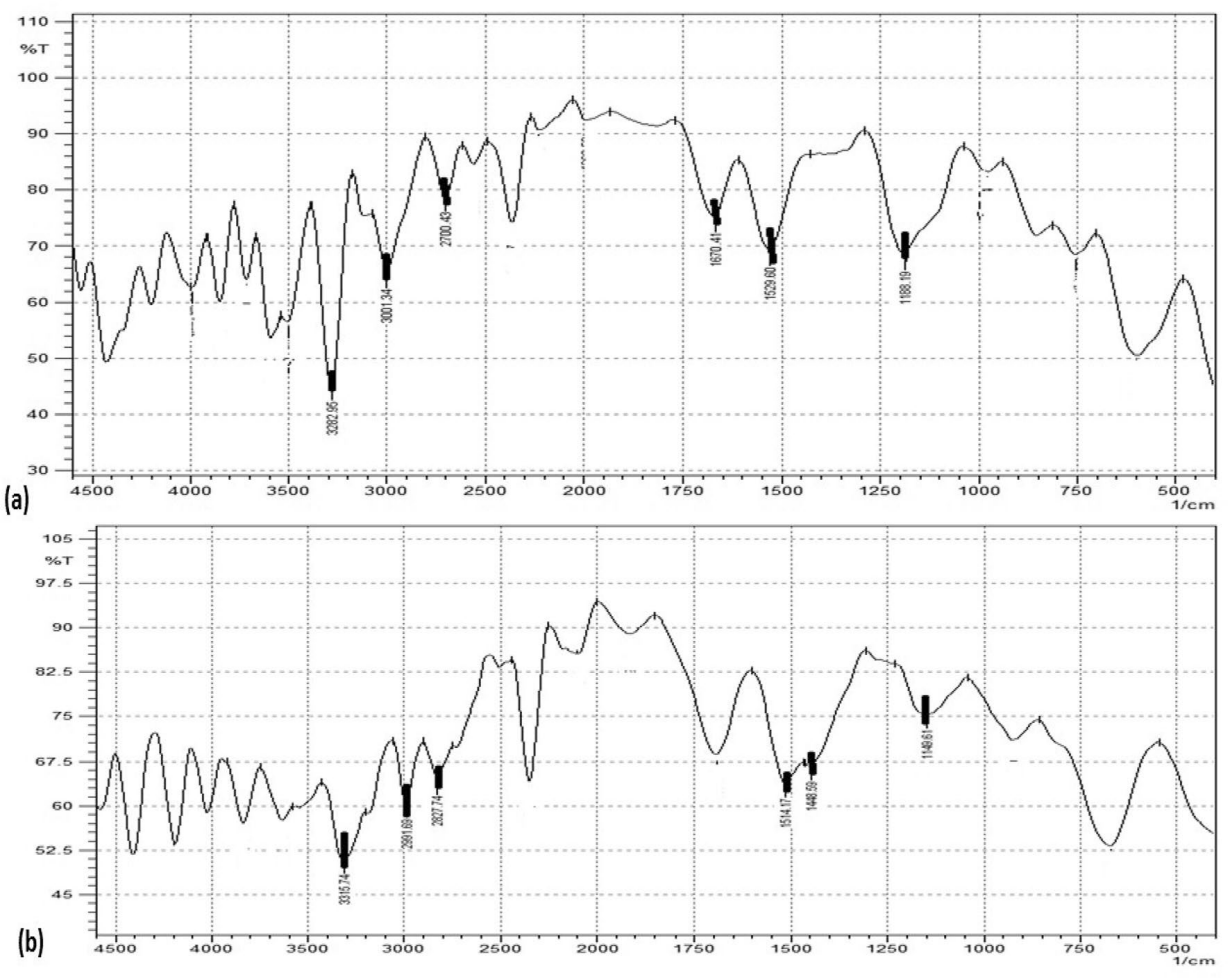
FTIR analysis subjected for 32 scans and scanning ranges from 4000–400 cm^-1^ using Shimadzu FTIR-8400S of (**a**) 3.5GPAMAM; (**b**) 4.0GPAMAM.

The Infrared (IR) spectrum of different generations of dendrimers was acquired. The IR spectrum of the synthesized dendrimer 4.0G showed absorption peaks at 3315.74 cm^−1^ for N–H stretching of primary amine, the peak at 2827.74 cm^−1^ for aliphatic C–H stretches, 1514.17 cm^−1^ and 1448.59 cm^−1^ peaks for N–H bending and 1149.61 cm^−1^ for C–C bending. A peak at 1654.5 cm^−1^ was assigned for amide carbonyl absorption while a peak at 1568.4 cm^−1^ was due to a core N–C stretching. The IR spectrum of the 3.5G showed absorption due to the presence of characteristic quaternary ammonium ion peak at 3282.95 cm^−1^ as well as carboxylic carbon at 1670.41 cm^−1^. Other peaks include 3001.34 cm^−1^ for N–H stretch, 2700.43 cm^−1^ for C-H stretch and 1188.19 cm^−1^ for C–C bending. Half generation carboxyl terminated shows intense peaks in the -C = O region while full generations show intense peaks in the -N–H stretch for primary amine. The appearance-disappearance reappearance of distinctive peaks stipulates the evidence of synthesis, as shown in Table2 and Fig. 7.

**Table 2:** IR interpretation of 4.0G and 3.5G PAMAM dendrimers.

S. No	Functional groups	Frequency (cm^-1^)
**4.0G PAMAM dendrimer**		
1	N–H stretch of primary amine	3315.74
2	N–H stretch anti-symmetric primary amine	2991.69
3	C-H stretch	2827.74
4	N–H bending of N-substituted amine	1514.17, 1448.59
5	C–C bending	1149.61
**3.5G PAMAM dendrimer**		
1	Quaternary ammonium ion peak	3282.95
2	N–H stretch anti-symmetric primary amine	3001.34
3	C–H stretch	2700.43
4	C = O stretch of carbonyl	1670.41
5	C–C bending	1188.19

### Solubility studies

The different generations of PAMAM dendrimers dissolved in solvents with varied solubility parameters were employed for the determination of solubility. The dissolvability of polymers is subjugated by internal energy interactions. The relative solvency of particular solvent, consequential of the solvent's cohesive energy density delineates the solubility parameter. Solubility reflects the degree of change in polymer composition. The studies were evaluated under steady and tumbling conditions. However, it was observed that even after tumbling for 36 h dendrimer solvency have significant effect by solvents with solubility parameter greater than 26.6 (MPa)^1/2^. Whereas, in both conditions negligible effect on solvency was observed on admixture of solvents with solubility parameter between 15.8–24.6 (MPa)^1/2^. The results in Table 3 clearly show the significant solubility with the incremental solubility parameter of solvents.

**Table 3:** Solubility of different generations of polyamidoamine dendrimer in some common solvents.

Solvents	Solubility parameter (MPa)^1/2^	PAMAM generation													
		1.0G		1.5G		2.0G		2.5G		3.0G		3.5G		4.0G	
		1	2	1	2	1	2	1	2	1	2	1	2	1	2
Diethyl ether	15.8	I	I	I	I	I	I	I	I	I	I	I	I	I	I
Cyclohexane	16.8	I	I	I	I	I	I	I	I	I	I	I	I	I	I
Chloroform	17.8	I	I	I	I	I	I	I	I	I	I	I	I	I	I
Toulene	18.2	I	I	I	I	I	I	I	I	I	I	I	I	I	I
Acetone	20.1	I	I	I	I	I	I	I	I	I	I	I	I	I	I
Acetic acid	21.3	I	I	I	I	I	I	I	I	I	I	I	I	I	I
Acetonitrile	24.6	I	I	I	I	I	I	I	I	I	I	I	I	I	I
Ethanol	26.6	S	S	S	S	S	S	S	S	S	S	S	S	S	S
Methanol	29.7	S	S	S	S	S	S	S	S	S	S	S	S	S	S
Ethylene glycol	32.9	S	S	S	S	S	S	S	S	S	S	S	S	S	S
Water	47.9	S	S	S	S	S	S	S	S	S	S	S	S	S	S

*1-Solubility 15 min after addition of solvent; 2-Solubility after 36 h of tumbling; *I* Insoluble *S* Soluble.

## Conclusion

Through a combined computational and experimental approach, a substantial endeavor has been dedicated for the synthesis of dendrimer. In this work we performed an extensive MD simulation on PAMAM dendrimers, with the purpose of elucidating several structural aspects. The effects associated with changes in pH (Fig. 4) are reflected by the electrostatic, and van der Waal forces. The work was pondered to develop minimal step, defect free, rapid and cost-effective synthesis. This study investigated the optimization and molecular dynamics as an approach to enhance the potency of molecules. The structural composition of dendrimer was successfully achieved via a divergent approach by reacting EDA and methyl acrylate in repeated steps. The synthesized dendrimer was characterized by UV–Vis, IR spectroscopy. For the assessment of morphological information UV–Vis spectroscopy was used. The Infrared spectroscopy was employed for the evaluation of chemical transits under way at the periphery of dendrimers, such as the disappearance of amine groups in the synthesis of half generation of dendrimers. The analysis data revealed synthesized dendrimer as suitable nanostructured candidate suitable for neutral as well as charged molecule delivery. Understanding the molecular dynamics as well as experimental studies governing the synthesis of dendrimer alludes that an interplay of essential parameters needs to be considered in order to achieve enhanced clinical application of dendrimers.

## Methods

### Material

Ethylenediamine (Merck Life Science Private Limited, Mumbai), Methyl acrylate (Central Drug House (P) Ltd., New Delhi), methanol (Merck Life Science Private Limited, Mumbai), Copper sulfate (Central Drug House (P) Ltd., New Delhi) were used. All the chemicals were used without further purification. The double-distilled water was used during all related studies.

### Determination of molecular dynamics

In an effort to retrieve knowledge about the molecular properties of dendrimer, the 3D simulations were carried out. The different generations of dendrimers were simulated using Avogadro software^60^ and further studied for the estimation of various molecular dimensions. In the exploration of molecular simulations, the force field act as an essential key element. The force field proportionally studies the estimation of potential energy and computation of forces acting on the atom, of the system under assessment. In this work, the polymeric dendritic molecules were parameterized employing the UFF i.e. Universal Forcefield. This forcefield is a broad spectra and non-reactive approach towards introduction of weighted atom types and weighted bonds, used to update topologies and atom parameterizations at every time step of a simulation. Each molecular model was then subjected to an amalgamation of steepest descent and conjugate gradient energy minimization steps of 25,000 cycles, in order to relax close atomic distances. The minimization methods steepest descent and conjugate gradient were employed in combination for effective results, where, former is faster and latter productive. Moreover, the system was equilibrated by performing 8–12 ns MD simulations in the isobaric-isothermal (NPT) ensemble. In the MD simulation, the atoms included in molecules move according to the Newtonian equation of motions. Subsequently, the 1 fs time step algorithm was employed to achieve the process of integrating the equation of motion, as integration method.

The resulting simulated molecules were subsequently energy minimized to yield van der Waals sphere at variable pH from 4.0 to 9.0 depicting electrostatic potential^59^. Equations used for the electrostatic interactions is Coulomb's law, and the equations used for the van der Waal's component is the 6–12 Lennard–Jones potential. Both type of interactions contributes to the adhesion of particles. For the ease of clarity 1.0G PAMAM generation was chosen here, as simulations of all generations are beyond the scope of this paper.

### Preparation of dendrimers

**Figure 8:**
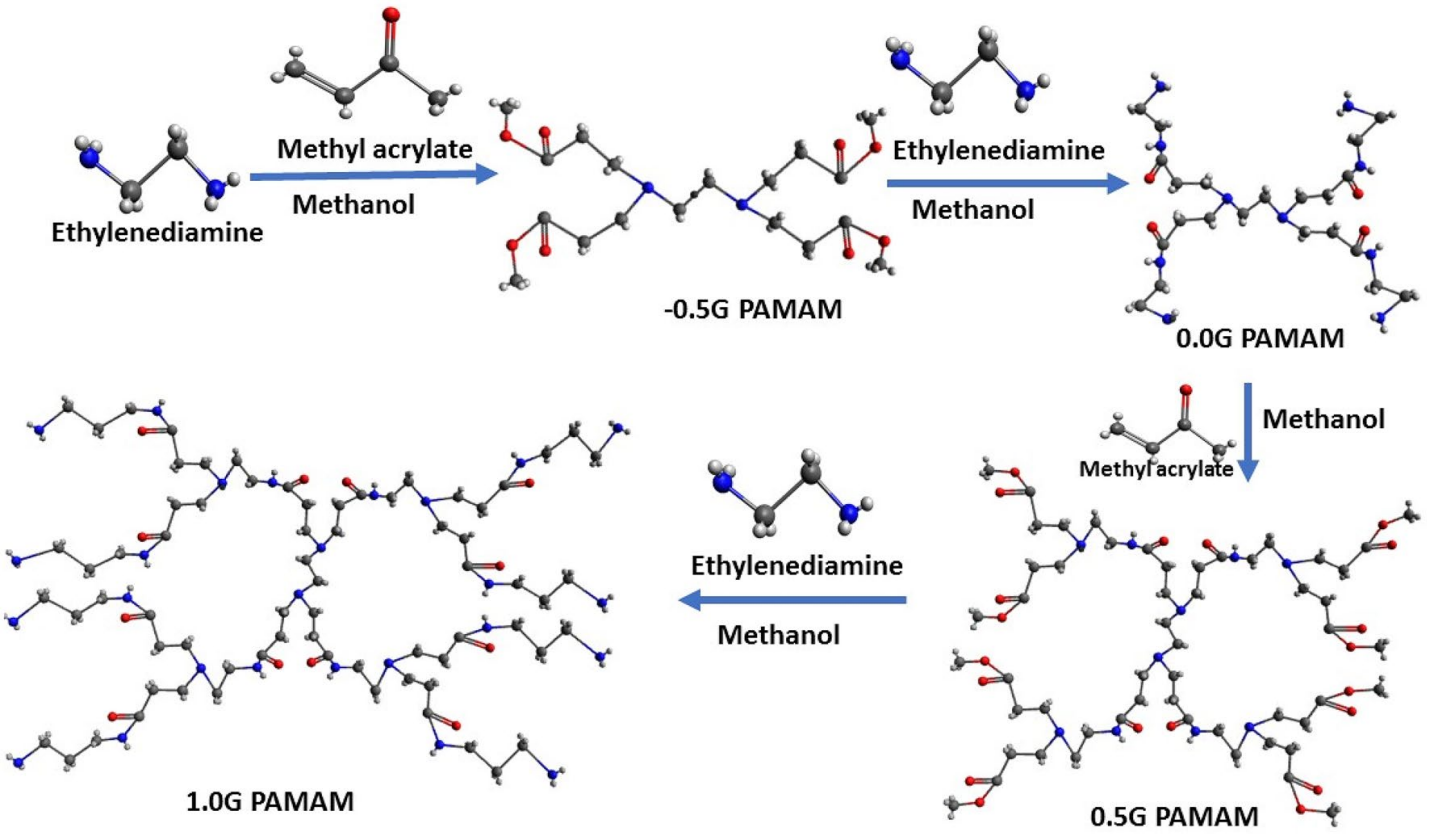
3D Molecular model illustrating the synthesis of PAMAM dendrimer (Blue: Nitrogen; Grey: Carbon; Red: Oxygen) (Avogadro 1.1.1, http://avogadro.cc).

The composition of dendrimers principally encompasses two steps; Michael addition and amidation. The reported method here involved ethylene diamine (EDA) as core, which was attached by acrylate resulting in −0.5G tetra ester. The next step followed the amidation of terminal carbomethoxy group by EDA. This tetra ester with an excess amount of EDA gave 0.0G tetra amine, Fig. 8. The process was repeated until the desired generation was achieved i.e. 4.0G. To circumvent the obstruction due to incomplete reaction, excess of EDA was used^61^. Excess of reagents was removed employing the usage of rotary vacuum evaporator maintained at 50–60 °C, in every step. All the reactions were advanced using tightly corked amber colored round bottom flasks obscured at room temperature for minimal step, defect-free, rapid and cost effective synthesis. The addition reaction took 2 days, whereas the amidation reaction took 4 days for the accomplishment of the array of reactions.

### Evaluation of dendrimers

The validation of synthesis of half and full generation dendrimers was determined by UV–Vis as well as FTIR spectroscopy. For the differentiation among half and full generation of dendrimer, 0.1% w/v dendrimers solution was stirred with freshly prepared 1% w/v aqueous solution of copper sulphate. To investigate the structural changes in dendrimer, FTIR spectroscopy was used. The samples were prepared in the form of KBr pellet method. It was subjected to 32 scans and scanning ranges from 4000–400 cm^−1^ using FTIR-8400S, Shimadzu, Kyoto, Japan.

### Solubility studies

Solubility parameters were determined according to the standardized American Society for Testing Materials (ASTM) D 3132–84 procedure with slight modifications^62^. In the experiment, 1 ml of 0.1 mg/ml each PAMAM dendrimers generations 1.0G, 1.5G, 2.0G, 2.5G, 3.0G, 3.5G and 4.0G along with solvents with varied solubility parameters were kept in test tubes. The test tubes were shaken at room temperature for 36 h in a shaker and allowed to stand to attain equilibrium. Visual inspections were conducted for the evaluation of solubility.

## References

[1] Azar NTP, Mutlu P, Khodadust R, Gunduz U (2013). Poly (amidoamine) (PAMAM) nanoparticles: synthesis and biomedical applications. J. Biol. Chem..

[2] Esfand R, Tomalia DA (2001). Poly(amidoamine) (PAMAM) dendrimers: from biomimicry to drug delivery and biomedical applications. Drug Discov. Today..

[3] Buhleier E, Wehner W, Vögtle F (1978). Cascade and nonskid-chain-like syntheses of molecular cavity topologies. Synthesis.

[4] Narayanan, V. V. & Newkome, G. R. Supramolecular chemistry within dendritic structures in *Dendrimers* (ed. Meijere, A.) 19–77 (Springer, Berlin, Heidelberg, 1998).

[5] Luostarinen D, Partanen T, Schalley CA, Rissanen K (2004). Synthesis of frechet-type resorcarene tetrabenzoxazine dendrimers. Synthesis.

[6] Hurst SK, Cifuentes MP, Humphrey MG (2002). A rapid convergent approach to organometallic dendrimers: sterically controlled dendron synthesis. Organometallics.

[7] McDonagh AM, Powell CE, Morrall JP, Cifuentes MP, Humphrey MG (2003). Convergent synthesis of alkynylbis (bidentate phosphine) ruthenium dendrimers. Organometallics.

[8] Kaushik RD, Singh J, Chawla M (2019). Dendritic polymers: An innovative step towards green future. Int. J. Pharm. Res..

[9] Singh J, Chawla M (2019). Dendritic polymers: a future approach in drug delivery system.

[10] Hobson LJ, Feast WJ (1999). Poly(amidoamine) hyperbranched systems: synthesis, structure and characterization. Polymer.

[11] Diaz C, Guzmán J, Jiménez VA, Alderete JB (2017). Partially PEGylated PAMAM dendrimers as solubility enhancers of Silybin. Pharm. Dev. Technol..

[12] Luong D, Kesharwani P, Killinger BA, Moszczynska A, Sarkar FH, Padhye S, Rishi AK, Iyer AK (2016). Solubility enhancement and targeted delivery of a potent anticancer flavonoid analogue to cancer cells using ligand decorated dendrimer nano-architectures. J. Colloid Interface Sci..

[13] Kesharwani P, Xie L, Banerjee S, Mao G, Padhye S, Sarkar FH, Iyer AK (2015). Hyaluronic acid-conjugated polyamidoamine dendrimers for targeted delivery of 3, 4-difluorobenzylidene curcumin to CD44 overexpressing pancreatic cancer cells. Colloids Surf. B Biointerfaces..

[14] Nabavizadeh F, Fanaei H, Imani A, Vahedian J, Amoli FA, Ghorbi J, Sohanaki H, Mohammadi SM, Golchoobian R (2016). Evaluation of nanocarrier targeted drug delivery of Capecitabine-PAMAM dendrimer complex in a mice colorectal cancer model. Acta Med. Iran..

[15] Liu Y, Ng Y, Toh MR, Chiu GN (2015). Lipid-dendrimer hybrid nanosystem as a novel delivery system for paclitaxel to treat ovarian cancer. J. Controlled Release..

[16] Wang T, Zhang Y, Wei L, Teng YG, Honda T, Ojima I (2018). Design, synthesis, and biological evaluations of asymmetric bow-tie PAMAM dendrimer-based conjugates for tumor-targeted drug delivery. ACS Omega..

[17] Zheng W, Cao C, Liu Y, Yu Q, Zheng C, Sun D, Ren X, Liu J (2015). Multifunctional polyamidoamine-modified selenium nanoparticles dual-delivering siRNA and cisplatin to A549/DDP cells for reversal multidrug resistance. Acta Biomater..

[18] Alibolandi M, Taghdisi SM, Ramezani P, Shamili FH, Farzad SA, Abnous K, Ramezani M (2017). Smart AS1411-aptamer conjugated pegylated PAMAM dendrimer for the superior delivery of camptothecin to colon adenocarcinoma in vitro and in vivo. Int. J. Pharm..

[19] Dichwalkar T, Patel S, Bapat S, Pancholi P, Jasani N, Desai B, Yellepeddi VK, Sehdev V (2017). Omega-3 fatty acid grafted PAMAM-paclitaxel conjugate exhibits enhanced anticancer activity in upper gastrointestinal cancer cells. Macromol. Biosci..

[20] Zhong Q, Bielski ER, Rodrigues LS, Brown MR, Reineke JJ, da Rocha SR (2016). Conjugation to poly (amidoamine) dendrimers and pulmonary delivery reduce cardiac accumulation and enhance antitumor activity of doxorubicin in lung metastasis. Mol. Pharm..

[21] Mekuria SL, Debele TA, Chou HY, Tsai HC (2015). IL-6 antibody and RGD peptide conjugated poly (amidoamine) dendrimer for targeted drug delivery of HeLa cells. J. Phys. Chem. B..

[22] Buczkowski A, Olesinski T, Zbicinska E, Urbaniak P, Palecz B (2015). Spectroscopic and calorimetric studies of formation of the supramolecular complexes of PAMAM G5-NH2 and G5-OH dendrimers with 5-fluorouracil in aqueous solution. Int. J. Pharm..

[23] Gupta L, Sharma AK, Gothwal A, Khan MS, Khinchi MP, Qayum A, Singh SK, Gupta U (2017). Dendrimer encapsulated and conjugated delivery of berberine: a novel approach mitigating toxicity and improving in vivo pharmacokinetics. Int. J. Pharm..

[24] Abdou EM, Masoud MM (2018). Gallic acid–PAMAM and gallic acid–phospholipid conjugates, physicochemical characterization and in vivo evaluation. Pharm. Dev. Technol..

[25] Gothwal A, Khan I, Kumar P, Raza K, Kaul A, Mishra AK, Gupta U (2018). Bendamustine–PAMAM conjugates for improved apoptosis, efficacy, and in vivo pharmacokinetics: a sustainable delivery tactic. Mol. Pharm..

[26] Xu X, Lü S, Gao C, Wang X, Bai X, Gao N, Liu M (2015). Facile preparation of pH-sensitive and self-fluorescent mesoporous silica nanoparticles modified with PAMAM dendrimers for label-free imaging and drug delivery. Chem. Eng. J..

[27] Najlah M, Freeman S, Khoder M, Attwood D, D’Emanuele A (2017). In vitro evaluation of third generation PAMAM dendrimer conjugates. Molecules.

[28] Choudhary S, Gupta L, Rani S, Dave K, Gupta U (2017). Impact of dendrimers on solubility of hydrophobic drug molecules. Front. Pharmacol..

[29] Patel J, Garala K, Basu B, Raval M, Dharamsi A (2011). Solubility of aceclofenac in polyamidoamine dendrimer solutions. Int. J. Pharm. Investig..

[30] Ertürk AS, Gürbüz MU, Tülü M (2017). The effect of PAMAM dendrimer concentration, generation size and surface functional group on the aqueous solubility of candesartan cilexetil. Pharm. Dev. Technol..

[31] Jose J, Charyulu RN (2016). Prolonged drug delivery system of an antifungal drug by association with polyamidoamine dendrimers. Int. J. Pharm. Investig..

[32] Hu Q, Chen Q, Yan X, Ding B, Chen D, Cheng L (2018). Chondrocyte affinity peptide modified PAMAM conjugate as a nanoplatform for targeting and retention in cartilage. Nanomedicine..

[33] Jeong SW, O’Brien DF, Orädd G, Lindblom G (2002). Encapsulation and diffusion of water-soluble dendrimers in a bicontinuous cubic phase. Langmuir.

[34] Zhou L, Russell DH, Zhao M, Crooks RM (2001). Characterization of poly (amidoamine) dendrimers and their complexes with Cu2+ by matrix-assisted laser desorption ionization mass spectrometry. Macromolecules.

[35] Pittelkow M, Moth- Poulsen K, Boas U, Christensen JB (2003). Poly (amidoamine)-dendrimer-stabilized Pd (0) nanoparticles as a catalyst for the Suzuki reaction. Langmuir.

[36] Ozturk O, Black TJ, Perrine K, Pizzolato K, Williams CT, Parsons FW, Ratliff JS, Gao J, Murphy CJ, Xie H, Ploehn HJ, Chen DA (2005). Thermal decomposition of generation-4 polyamidoamine dendrimer films: decomposition catalyzed by dendrimer-encapsulated Pt particles. Langmuir.

[37] Zarebkohan A, Najafi F, Moghimi HR, Hemmati M, Deevband MR, Kazemi B (2015). Synthesis and characterization of a PAMAM dendrimer nanocarrier functionalized by SRL peptide for targeted gene delivery to the brain. Eur. J. Pharm. Sci..

[38] Najafi F, Moghimi HR, Hemmati M, Deevband MR, Kazemi B (2016). SRL-coated PAMAM dendrimer nano-carrier for targeted gene delivery to the glioma cells and competitive inhibition by lactoferrin. Iran J. Pharm. Res..

[39] Alnasser Y, Kambhampati SP, Nance E, Rajbhandari L, Shrestha S, Venkatesan A, Kannan RM, Kannan S (2018). Preferential and increased uptake of hydroxyl-terminated PAMAM dendrimers by activated microglia in rabbit brain mixed glial culture. Molecules.

[40] Vögtle F, Reichardt G (2007). Werner.

[41] Bellini RG, Guimarães AP, Pacheco MA, Dias DM, Furtado VR, de Alencastro RB, Horta BA (2015). Association of the anti-tuberculosis drug rifampicin with a PAMAM dendrimer. J. Mol. Graph. Model..

[42] De Luca S, Seal P, Ouyang D, Parekh HS, Kannam SK, Smith SC (2016). Dynamical interactions of 5-fluorouracil drug with dendritic peptide vectors: the impact of dendrimer generation, charge, counterions, and structured water. J. Phys. Chem. B..

[43] Zhang FD, Liu Y, Xu JC, Li SJ, Wang XN, Sun Y, Zhao XL (2015). Binding and conformation of dendrimer-based drug delivery systems: A molecular dynamics study. Adv. Manuf..

[44] Rengaraj A, Subbiah B, Haldorai Y, Yesudhas D, Yun HJ, Kwon S, Choi S, Han YK, Kim ES, Huh YS (2017). Correction: PAMAM/5-fluorouracil drug conjugate for targeting E6 and E7 oncoproteins in cervical cancer: a combined experimental/in silico approach. RSC Adv..

[45] Ouyang D, Zhang H, Parekh HS, Smith SC (2011). The effect of pH on PAMAM dendrimer-siRNA complexation: Endosomal considerations as determined by molecular dynamics simulation. Biophys. Chem..

[46] Lim J, Lo ST, Hill S, Pavan GM, Sun X, Simanek EE (2012). Antitumor activity and molecular dynamics simulations of paclitaxel-laden triazine dendrimers. Mol. Pharm..

[47] Schneider CP, Shukla D, Trout BL (2011). Effects of solute-solute interactions on protein stability studied using various counterions and dendrimers. PLoS ONE.

[48] Kelly CV, Leroueil PR, Orr BG, Banaszak Holl MM, Andricioaei I (2008). Poly(amidoamine) dendrimers on lipid bilayers II: Effects of bilayer phase and dendrimer termination. J. Phys. Chem. B..

[49] Mills M, Orr BG, Banaszak Holl MM, Andricioaei I (2013). Attractive hydration forces in DNA-dendrimer interactions on the nanometer scale. J. Phys. Chem. B..

[50] Filipe CS, Machuqueiro M, Darbre T, Baptista M (2013). Unraveling the conformational determinants of peptide dendrimers using molecular dynamics simulations. Macromolecules.

[51] Wang Y-L, Lu Z-Y, Laaksonen A (2012). Specific binding structures of dendrimers on lipid bilayer membranes. Phys. Chem. Chem. Phys..

[52] Tian W, Ma Y (2012). Insights into the endosomal escape mechanism via investigation of dendrimer–membrane interactions. Soft Matter.

[53] Zhong T, Ai P, Zhou J (2011). Structures and properties of PAMAM dendrimer: a multi-scale simulation study. Fluid Phase Equilib..

[54] Quintana A, Raczka E, Piehler L, Lee I, Myc A, Majoros I, Patri AK, Thomas T, Mulé J, Baker JR (2002). Design and function of a dendrimer-based therapeutic nanodevice targeted to tumor cells through the folate receptor. Pharm. Res..

[55] Shi X, Lee I, Chen X, Shen M, Xiao S, Zhu M, Baker JR, Wang SH (2010). Influence of dendrimer surface charge on the bioactivity of 2-methoxyestradiol complexed with dendrimers. Soft Matter.

[56] Stach M, Maillard N, Kadam RU, Kalbermatter D, Meury M, Page MGP, Fotiadis D, Darbre T, Reymond JL (2012). Membrane disrupting antimicrobial peptide dendrimers with multiple amino termini. Med. Chem. Comm..

[57] Al-Jamal KT, Al-Jamal WT, Wang JT-W, Rubio N, Buddle J, Gathercole D, Zloh M, Kostarelos K (2013). Cationic poly-L-lysine dendrimer complexes doxorubicin and delays tumor growth in vitro and in vivo. ACS Nano.

[58] Liu Y, Bryantsev VS, Diallo MS, Goddard WA (2009). PAMAM dendrimers undergo pH responsive conformational changes without swelling. J. Am. Chem. Soc..

[59] Evangelista-Lara A, Guadarrama P (2005). Theoretical evaluation of the nanocarrier properties of two families of functionalized dendrimers. Int. J. Quantum Chem..

[60] Marcus D, Hanwell DE, Curtis DC, Lonie TV, Zurek E, Hutchison GR (2012). Avogadro: an advanced semantic chemical editor, visualization, and analysis platform. J. Cheminformatics..

[61] Tomalia DA, Baker H, Dewald JR, Hall M, Kallos G, Martin S, Roeck J, Ryder J, Smith P (1986). Dendritic macromolecules: synthesis of starburst dendrimers. Macromolecules.

[62] Uppuluri, S., Dvornic, P. R., Klimash, J. W., Carver, P. I., & Tan, N. C. B. The properties of Dendritic polymers I: Generation 5 Poly(amidoamine) dendrimers. (Army Research Laboratory 1998).

